# DNA Barcoding to Confirm the Morphological Identification of the Coral Trees (*Erythrina* spp., Fabaceae) in the Ancient Gardens of Naples (Campania, Italy)

**DOI:** 10.3390/plants7020043

**Published:** 2018-06-06

**Authors:** Adriana De Luca, Giancarlo Sibilio, Paolo De Luca, Emanuele Del Guacchio

**Affiliations:** 1 Dipartimento di Medicina Veterinaria e Produzioni Animali, Università degli Studi di Napoli Federico II, Via Delpino 1, 80137 Napoli, Italy; adriana.deluca@unina.it; 2 Botanical Garden of Naples, Università degli Studi di Napoli Federico II, Via Foria 223, 80139 Napoli, Italy; pdeluca@unina.it (P.D.L.); edelgua@email.it (E.D.G.)

**Keywords:** botanical garden, botanical history, Dehnhardt, DNA barcoding, urban gardens

## Abstract

The coral trees (genus *Erythrina*) have been fostering great interest among the botanists and gardeners of Naples, since their arrival in Europe in the second half of the 18th century. Numerous species were present in the royal and private botanical gardens of the region, but their number has decreased today. The purpose of this work was to verify which species occur nowadays in the public areas of Naples and associate them with the historical information about their introduction. The identification was carried out also by molecular methods, by means of sequencing nuclear and chloroplast DNA markers. The comparison of the sequences obtained for the specimens present in Naples with those present in the literature, together with a morphological examination, allowed us to identify with accuracy the species anciently introduced or nowadays cultivated in Naples.

## 1. Introduction

Genus *Erythrina* L. (Fabaceae) consists of about 120 species [1,2,3], mostly trees and shrubs. They are widespread especially in the tropical areas, with the highest number of taxa in America and a smaller number in Africa and Asia [4]. They are most prominently used for ornamental purposes in the warm areas of the world [5], where they become naturalized in some cases [6,7].

In Europe, *Erythrina* species were originally cultivated mainly in greenhouses. *Erythrina abyssinica* Lam. arrived in Europe in 1773 following the journeys of J. Bruce in Africa [8]. *Erythrina crista-galli* L. arrived in Italy in 1772, at the Botanical Garden of Turin, while *Erythrina americana* Mill. (as *Erythrina coralloides* DC.) was introduced into the Garden of Lady Durazzo Grimaldi in Genoa in 1805 [9].

In the “Gordon, Dermer, and Thomson” catalog [10], *Erythrina herbacea* L., *Erythrina corallodendron* L. and *Erythrina picta* L. were reported (the latter without indication of the author). In the Baumann Brothers’ Catalog [11], published in Germany and France, “*Erythrina capensis*” (an unidentified species), *E. corallodendron* L., and *Erythrina variegata* L. (sub *E. picta* L.) were available for sale. These catalogues suggest that the plants were present in many collections in Europe at that time. In the second half of the 19th century, at the Botanical Garden of Palermo, the following species were recorded: *Erythrina insignis* Tod., probably previously cultivated in the nearby Botanical Garden of “Bocca di Falco”; *E. crista-galli* L. (under the name *Erythrina pulcherrima* Tod. [12]), probably introduced by seeds from Egypt, and *Erythrina arborescens* Roxb. (as *Erythrina moori* Tod. [12]), imported from India. In Palermo, the trees of *Erythrina caffra* Thunb., planted in the same period, are still living [13].

In the Kingdom of Naples, the presence of the genus dates back to 1803, in the Royal Park of Caserta [14]. John Graefer (or Graeffer; Helmstedt, 1746–Bronte, 1802), superintendent of the gardens, reported two species: *E. corallodendron* and *E. picta*, either without an author’s indication. He very likely obtained these species by the Thomson & Gordon nurseries, where he had been working earlier [15,16]. However, no *Erythrina* species was reported by Nicola Terracciano [17] in its description of the rare plants growing in the gardens of Caserta, and nowadays this genus is not cultivated there (G.S., pers. obs.).

No escaped individual has been reported for Italy [18] or Campania [19], because of the reproductive difficulties in our climates and of the relative rarity of *Erythrina* plants in the Italian gardens as well.

This work can be considered as part of a research project on the origin of the floristic diversity of the parks and gardens of Naples [20,21,22,23,24,25,26]. The purpose of the present contribution is to verify the occurrence of the genus *Erythrina* in the main public gardens of Naples and to attempt a reconstruction of the history of their introduction into the city. 

## 2. Material and Methods

Historical information was retrieved by consulting the plant catalogues and *Indices seminum* of the Botanical Garden of Naples, the Royal Garden of Caserta, the *Hortus Camaldulensis*, and the private botanical garden of the Villa ‘Bisignano’ [14,17,27,28,29,30,31,32,33,34,35,36,37,38,39,40,41,42,43].

The State Archives of Naples, a relevant source of documents for the history of the cultivated flora of Naples, were investigated as well.

Precious information was obtained from the examination of the dried specimens preserved at the *Herbarium Neapolitanum* hosted at the Botanical Garden of Naples, where we found pertinent samples in the collections of the eminent botanists Michele Tenore (Napoli, 1780–1861) and Giovanni Gussone (Villamaina 1787–Napoli 1866).

Various species mentioned in the catalogues lack both an author name and an accurate description that would allow species identification. Indeed, in several cases, we found *nomina nuda* (i.e., names lacking any valid description and therefore not accepted by the ‘International Code of Nomenclature for algae, fungi, and plants’), such as “*Erythrina pisonaia*” [38] or “*Erythrina bellengeri*” [43], with the impossibility of accurately establishing the botanical species mentioned. Field researches aimed at mapping the presence of *Erythrina* were carried on in the most important historical parks and gardens of the administrative territory of Naples municipality. The identification was carried out using several floras and monographs [1,7,44,45,46,47]. 

For the purpose of confirming the identity of morphologically dubious or sterile individuals, the leaves from some individuals planted in Naples (see Table 1) were collected and analyzed by molecular techniques (DNA barcoding). In addition, for purposes of comparison and verification, one specimen of *E. caffra* obtained from South Africa, one specimen of *E. americana* (once present in the city) originating from Mexico, and one of *Erythrina latissima* (for which *mat*K was not available in the literature), cultivated by seeds collected in the wild (South Africa), were included in the molecular analysis. Among the available molecular markers, nuclear DNA ITS2 and chloroplast DNA *rbc*L and *mat*K were chosen for the analysis. The genomic DNAs were isolated from young leaves using the protocol by Doyle & Doyle [48]; PCR amplifications and sequencing were carried out according to De Luca et. al. [49], with the exception of the PCR conditions, which were as follows: initial denaturation at 95 °C for 5 min, followed by 35 denaturation cycles at 95 °C for 45 s, annealing at 55 °C for 45 s, extension at 72 °C for 1 min, and a final extension at 72 °C for 3 min. The raw sequences were analyzed through the Bio Edit software [50], and the identification of sequence barcodes from the samples was conducted using the Basic Local Alignment Search Tool (BLAST (NCBI, Bethesda, MD, USA); [51]).

In order to have a broad picture of the phylogenetic position in the genus of the species presently cultivated in Naples, as well as of *E. americana*, which was not available in the literature, we carried out a Bayesian analysis as well. 

The selection was carried out by choosing only those taxa for which both *mat*K and *rbc*L sequences were available. ITS2 sequences were not employed, given the small number of *Erythrina* taxa available in the literature for this marker (overall, less than 10 taxa are available for all three markers). When various accessions for the same taxon were identical in sequence, only one was selected; only *Erythrina humeana*, for which two different sequences were available for one marker (see Table 2), was employed with two separate accessions. Sequences of *Dysolobium grande* (Wall. ex Benth.) Prain were employed as outgroups. Such strategy resulted in the selection of the 24 sequences indicated in Table 2. All sequences were aligned by using ClustalW [52] as implemented in Bioedit [50] ver. 9.2. Separate alignments were then reduced to the same length of the regions obtained in this paper. The aligned sequences were then investigated through Bayesian analysis, by using the MrBayes ver. 3.1.2 software [53]. The most likely substitution models were separately computed by using the jModeltest ver. 2.1.7 software [54]. Then, a partitioned matrix was prepared, and four Markov chains (three hot, one cold) were run for 2,000,000 generations, under a GTR + G substitution model [55,56] for *mat*K and a K80 model [57] for *rbc*L. The taxonomic treatment followed the monography about the genus *Erythrina* by Krukoff & Barneby [1], with the updated nomenclature by the database Tropicos [58]. 

## 3. Results

### 3.1. Historical Sources

The first species to be reported in the Capital was *E. herbacea* [27], listed in the first catalogue of the ancient botanical garden of Prince Sanseverino di Bisignano in Barra (Napoli 1790–Roma 1865), a suburb of Naples. Later, several *Erythrina* were introduced by the German gardener and botanist Friedrich Dehnhardt (Bühle, 1787–Napoli, 1870) into the *Hortus Camaldulensis*, an important private garden [19,25,59]: *E. americana* (under the synonym *Erythrina laeta* Dehnh.), *E. corallodendron*, *E. herbacea*, and *Erythrina speciosa* Andrews (by the name ‘*E. Gräfferi’*) [41,42]. Unfortunately, both the garden of Camaldoli and that of Prince Bisignano disappeared long time ago.

The *Erythrina* species mentioned in the above cited works, as well as those occurring in the *Index Seminum* and in the catalogue of the Botanical Garden of Naples, are listed in Table 3. 

The consultation of the material preserved in the State Archives of Naples attested further introductions of *Erythrina* species in Naples. An 1833 document reports that F. Dehnhardt proposed the introduction of “*E. coralloides”* (=*E. americana*) [60] to adorn the Virgil’s Temple at Villa di Chiaja. In a document dated 1839, *E. crista-galli*, “*E. longifolia*” (nomen nudum), and *E. corallodendron* [60] are mentioned among the plants to be used for the flowerbeds of the Villa, which were called “Flora” and “Boschetto”. In 1844, a document listing the plants of the Villa Reale reports “*Erythrina laurifolia”* (=*E. crista-galli*), [61]. Finally, another document (concerning the years 1856–1859) signed by Dehnhardt is a list of plants to be bought for the villa, generically including *Erythrina* plants [62]. Further information can be found in Pasquale [63], who cited again “*E. laurifolia”* (=*E. crista-galli*) for the first flowerbeds of the Villa and *E. corallodendron* for the Temple of Virgil. In another article, Pasquale [64] reports that the rare coral tree of *E. corallodendron* bloomed in the Villa every year.

This latter species is also reported by G. Aiello [65] in describing the Flora of Naples. He writes about outdoor cultivated individuals of *E. corallodendron* on the Vomero hill.

### 3.2. Herbaria Specimens

In the *Herbarium Neapolitanum* (herbarium code: NAP), specimens of *Erythrina* were found in the herbaria of Gussone (Collection “Generale”) and in that of Tenore (Appendix A). The morphological examination of this material and the comparison allowed us to state that, at least in some cases, the name *E. corallodendron* was misapplied by local botanists. In fact, the specimens labelled as *E. corallodendron* are to be referred instead to *E. caffra*, while those labelled as *E. speciosa* represent a variation without taxonomical importance of *E. crista-galli.* Thus, the herbaria specimens can prove the cultivation, at that time, only of the following species: *E. caffra*, *E. crista-galli*, and *E. herbacea*. 

### 3.3. Erythrina Plants in the Gardens of Naples at Present

**Figure 1:**
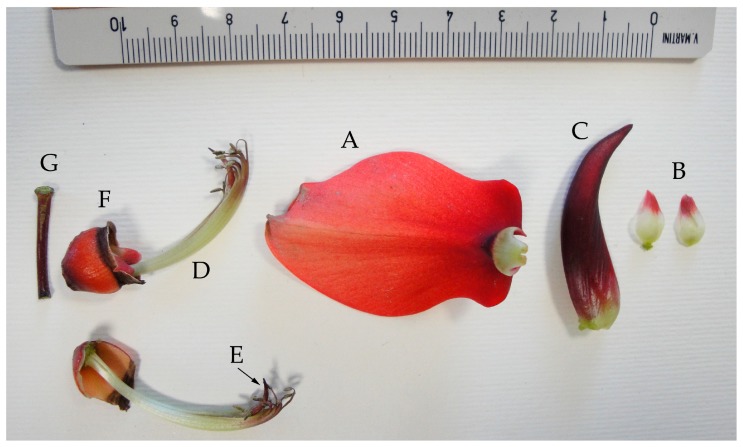
Dissected flowers of *Erythrina crista-galli* L., collected at Piazza Vittoria, Naples. Legend: (**A**) standard; (**B**) wing; (**C**) keel; (**D**) staminal tube; (**E**) style; (**F**) calyx; (**G**) pedicel.

**Figure 2:**
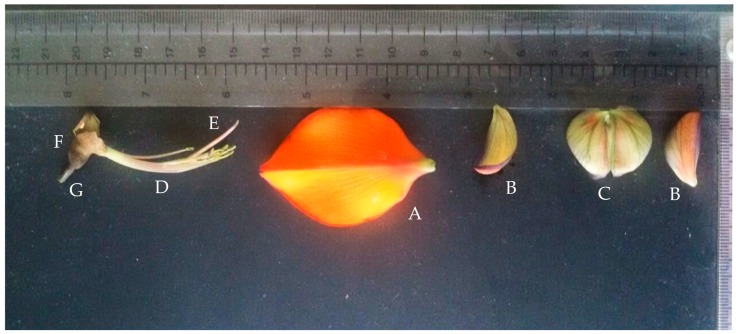
Dissected flower of the “*Erythrina* of Dehnhardt” (i.e., *E. caffra* Thunb.) at the Botanical Garden of Naples. Legend: (**A**) standard; (**B**) wing; (**C**) keel; (**D**) staminal tube; (**E**) style; (**F**) calyx; (**G**) pedicel.

**Figure 3:**
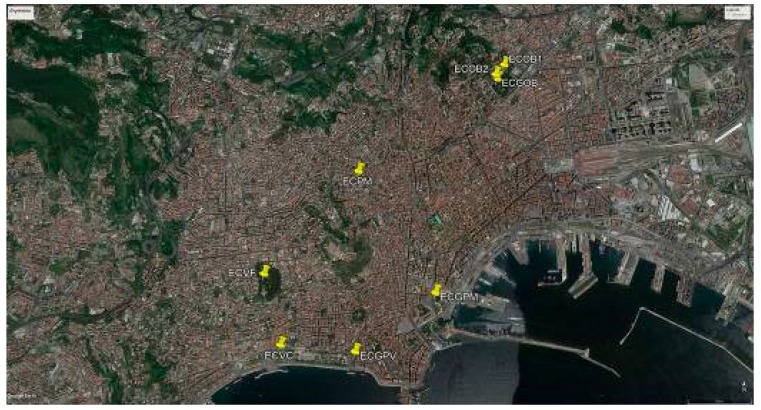
*Erythrina* specimens growing in the city of Naples, image prepared by using Google Earth cartography. Refer to Table 4 for the legend of the points.

We located 14 individuals of genus *Erythrina* cultivated in six public areas of Naples. All the studied *Erythrina* plants are to be referred only to *E. crista-galli* or *E. caffra.* The former species has been mainly identified by the following features: (1) calyx tube shallowly campanulate and glabrous or almost so; (2) keel obliquely lanceolate, longer than half of the standard; (3) standard contracted at the base into a reduced claw; (4) wings minute and much shorter than the keel; (5) inflorescences terminal and leafy or axillary; (6) staminal filaments free only toward the apex (for up to 7 mm); (7) corolla scarlet (see Figure 1 for the flower details). The other species, i.e., *E. caffra*, has been mainly identified by the following features: (1) calyx with cylindrical tube and not-bilabiate in bud but after bilabiate, and pubescent; (2) standard broadly ovate and arcuate; (2) keel petals united by their exterior margin; (3) keel a little shorter than the wings; (4) keel petals not acuminate; (5) wings obtuse; (6) corolla orange-red (see Figure 2 for the flower details). (A) At the Botanical Garden of Naples, an *Erythrina* tree 10 m high is present in the collections, in addition to a younger individual originated from it. This plant is locally called the “Dehnhardt tree” and is labelled as “*E. laeta* Dehnh.”. The morphological analysis allowed to identify this tree as *E. caffra*, and molecular investigations confirmed this as well. In addition, several *E. crista-galli* individuals are cultivated there. (B) In the area of the Villa Comunale (Villa Reale), there are also six individuals attributable to *E. crista-galli* (Piazza Vittoria and Piazza dei Martiri) and one belonging to *E. caffra*, in the exact site cited by Dehnhardt in 1833 [60] and Pasquale [63] under the name “*E. corallodendron”.* (C) In the Royal Park of Capodimonte and at (D) Villa Floridiana, we found two individuals attributable to *E. caffra* but only doubtfully, as they were without flowers at the gathering time. Their identity was confirmed by barcoding. (E) Two individuals of *E. crista-galli* were planted in the flowerbeds of Piazza Municipio. (F) Finally, a 12 m tall specimen of *E. caffra* can be observed in Piazza Mazzini. Table 4 reports the distribution of the sites throughout the city of Naples and their coordinates in UTM (Universal Transverse Mercator) extracted from Google Earth. Figure 3 shows the location on the map of Naples.

### 3.4. Genetic Analysis

The genetic analysis confirmed, after the morphological identification, that all the *Erythrina* plants at present cultivated in the gardens of Naples are to be referred only to *E. crista-galli* or *E. caffra* (the sample of *E. caffra* of Villa Comunale was considered identical, after systematic analysis, to the other samples of *E. caffra* and, for this reason, not included in the molecular investigation). The percentage of identity between the sequences of *E. crista-galli* obtained here and the corresponding ITS2, *mat*K, and *rbc*L sequences in the literature for the same species was 99–100% (Table 1). For *E. caffra*, the percentage of identity between our sequences and the corresponding *mat*K and *rbc*L sequences from the literature also was 99–100%. ITS2 sequences of *E. caffra* are not available in the literature; the highest percentage of identity of our ITS2 sequences was with *Erythrina velutina* (94%, Table 1).

The sequence details are shown in the Appendix B. For the specimen codes, see Table 1, the specimen of *E. caffra* obtained by the Manie van der Schijff Botanical Garden (Pretoria, South Africa) collections has been used as a control.

**Figure 4:**
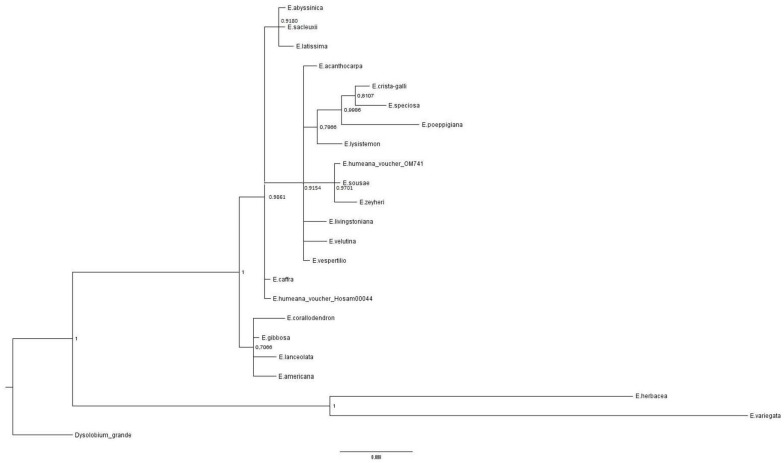
The Bayesian analysis was fully convergent at 2,000,000 generations and all estimated sample sizes were >>100.

The Bayesian analysis was fully convergent at 2,000,000 generations, and all Estimated Sample Sizes were >>100. The 95% maximum clade probability tree (Figure 4) showe wide collapses, but our species of interest, i.e., *E. caffra* and *E. crista-galli* (and *E. americana* as well), could be recovered in different clades: *E. crista-galli* was included in a clade with *E. speciosa* (which is its sister group), *Erythrina poeppigiana*, and *Erythrina lysistemon* (posterior probability p.p. = 0.7866); *E. caffra* was in a central collapse in the phylogram together with *Erythrina humeana* (voucher Hosam 00044), whereas *E. americana*, was included in a clade with *E. corallodendron*, *Erythrina gibbosa,* and *Erythrina lanceolata*, even if with a quite low posterior probability (*p* = 0.7066). *E. latissima*, for which *mat*K sequence was obtained here, is in a monophyletic group together with *E. abyssinica* and *Erythrina sacleuxii* (*p* = 0.9180).

## 4. Discussion and Conclusions

Despite only *E. crista-galli* and *E. caffra* are found in cultivation in the public areas of Naples nowadays, historical researches indicate the presence of other species, such as *E. americana* and *E. herbacea*. It is therefore possible that historical plants, belonging to delicate species, died and were later replaced by more robust ones. This hypothesis is supported by the young age of the plants at Villa Floridiana and Capodimonte. However, a doubt remains on whether *E. corallodendron* was effectively cultivated in the parks of Naples. The absence of accurate descriptions does not help in this respect. Pasquale [37] cited both *E. corallodendron* and *E. caffra*, and these two species obviously can be easily separated during identification. Surprisingly, the examination of historical specimens collected by Gussone and Pasquale at the Villa Reale proves that *E. corallodendron* was a misapplied name for *E. caffra*. In addition, the “*E. laeta*” of the Botanical Garden of Naples (ECOB1 and ECOB2) was found to be actually *E. caffra* itself, not *E. americana*, which is the accepted name for *E. laeta* [25]. Referring now to the nomen nudum “*Erythrina andersonii*” (also reported as “*E. crista-galli* var. *andersonii*”), which was employed by local botanists (Tenore and Gussone) and presumably in horticulture, the examined specimens labelled by that name result to belong to a broad-leaved form *E. crista-galli*, not worth of taxonomical recognition. Besides, the “*Erythrina speciosa*”, cultivated in the early 19th century in Caserta [14], is *E. crista-galli* as well, as annotated by Gussone in the labels of his collection. In Naples, only *E. crista-galli* appears as fully acclimatized, producing intense and vivid blossoms. On the contrary, *E. caffra* blooms only sporadically over the years and suffers from occasional frosts in the winters. It is indeed much more rarely cultivated than the former species.

This contribution on the historical presence of *Erythrina* species introduced as ornamental trees in the Kingdom of Naples would have not been possible without the combined usage of morphological identification methods, herbarium and archival research, and DNA barcoding. All these joined techniques, in fact, allowed us to detect first introductions, early misapplication of names, and the present reduction in biodiversity of the cultivated species. A multidisciplinary approach, which includes a mixture of classical and more recent methods in a coherent research strategy, is often the key in reconstructing the history of the introduction of alien plants.

## Figures and Tables

**Table 1:** Samples employed in the present study. *Legend: ECOB: Erythrina caffra* of the Botanical Garden of Naples; *ECSA: E. caffra* of the Manie van der Schijff Botanical Garden (Southafrica); ECPM: *E. caffra* of “Piazza Mazzini”, Naples; ECVF: *E. caffra* of “Villa Floridiana”, Naples; ECGPV: *Erythrina crista-galli* of “Piazza Vittoria”, Naples; ECGPM: *E. crista-galli* of “Piazza Municipio”, Naples; ECGOB: *E. crista-galli* of Naples Botanical Garden. The column “Genbank no.” indicates the accession number of the literature sequence with which the highest identity was verified.

Code	Taxon	Origin	Identities	Description2	GenBank no.
ECOB	*Erythrina caffra*	Botanical Garden, Naples, Italy (2016)	293/313 (94%)	*Erythrina velutina*	JX856571 (ITS2)
ECSA	*Erythrina caffra*	Manie van der Schijff Botanical Garden, Petroria, South Africa (2016)	293/313 (94%)	*Erythrina velutina*	JX856571 (ITS2)
ECVF	*Erythrina caffra*	Villa Floridiana, Naples, Italy (2016)	294/313 (94%)	*Erythrina velutina*	JX856572 (ITS2)
ECPM	*Erythrina caffra*	Piazza Mazzini, Naples, Italy (2016)	293/313 (94%)	*Erythrina velutina*	JX856571 (ITS2)
ECCPV	*Erytryna crista-galli*	Piazza Vittoria, Naples, Italy (2016)	311/312 (99%)	*Erythrina crista-galli*	FN825780 (ITS2)
ECCPM	*Erytryna crista-galli*	Piazza Municipio, Naples, Italy (2016)	311/312 (99%)	*Erythrina crista-galli*	FN825780 (ITS2)
ECGOB	*Erytryna crista-galli*	Botanical Garden, Naples, Italy (2016)	312/312 (100%)	*Erythrina crista-galli*	FN825781 (ITS2)
ECOB	*Erythrina caffra*	Botanical Garden, Naples, Italy (2016)	497/497 (100%)	*Erythrina caffra*	JQ412236 (*mat*K)
ECSA	*Erythrina caffra*	Manie van der Schijff Botanical Garden, Petroria, South Africa (2016)	497/497 (100%)	*Erythrina caffra*	JQ412236 (*mat*K)
ECPM	*Erythrina caffra*	Villa Floridiana, Naples, Italy (2016)	496/499 (99%)	*Erythrina caffra*	JQ412236 (*mat*K)
ECVF	*Erythrina caffra*	Piazza Mazzini, Naples, Italy (2016)	495/499 (99%)	*Erythrina caffra*	JQ412236 (*mat*K)
ECGPV	*Erythrina crista-galli*	Piazza Vittoria, Naples, Italy (2016)	511/514 (99%)	*Erythrina crista-galli*	AY386869 (*mat*K)
ECGPM	*Erythrina crista-galli*	Piazza Municipio, Naples, Italy	511/514 (99%)	*Erythrina crista-galli*	AY386869 (*mat*K)
ECGOB	*Erythrina crista-galli*	Botanical Garden, Naples, Italy	510/514 (99%)	*Erythrina crista-galli*	AY386869 (*mat*K)
ECOB	*Erythrina caffra*	Botanical Garden, Naples, Italy (2016)	469/469 (100%)	*Erythrina caffra*	JQ412356 (*rbc*L)
ECSA	*Erythrina caffra*	Manie van der Schijff Botanical Garden, Petroria, South Africa (2016)	469/469 (100%)	*Erythrina caffra*	JQ412356 (*rbc*L)
ECPM	*Erythrina caffra*	Villa Floridiana, Naples, Italy (2016)	469/469 (100%)	*Erythrina caffra*	JQ412356 (*rbc*L)
ECVF	*Erythrina caffra*	Piazza Mazzini, Naples, Italy (2016)	469/469 (100%)	*Erythrina caffra*	JQ412356 (*rbc*L)
ECGPV	*Erythrina crista-galli*	Piazza Vittoria, Naples, Italy (2016)	505/508 (99%)	*Erythrina crista-galli*	Z70170 (*rbc*L)
ECGPM	*Erythrina crista-galli*	Piazza Municipio, Naples, Italy	503/508 (99%)	*Erythrina crista-galli*	Z70170 (*rbc*L)
ECGOB	*Erythrina crista-galli*	Botanical Garden, Naples, Italy	505/508 (99%)	*Erythrina crista-galli*	Z70170 (*rbc*L)

**Table 2:** Sequences employed for the Bayesian Inference investigation.

Taxon	Genbank Acc. No.	
	*mat*K	*rbc*L
*Erythrina abyssinica* Lam.	JX518054	JX572563
*Erythrina americana* Mill.	This paper	This paper
*Erythrina acanthocarpa* E.Mey.	KF147397	KF147471
*E. caffra* Thunb.	JQ412236	JQ412356
*Erythrina corallodendron* L.	KJ012577	KJ082284
*E. crista-galli* L.	AY386869	Z70170
*Erythrina gibbosa* Cufod.	JQ587632	JQ591749
*Erythrina herbacea* L.	KJ772770	KJ773492
*Erythrina humeana* Spreng. (voucher Hosam 00044)	JX495709	JX571824
*E. humeana* (voucher OM741)	JF270763	JF265413
*Erythrina lanceolata* Standl.	JQ587635	JQ591753
*Erythrina latissima* E.Mey.	This paper	JF265414
*Erythrina livingstoniana* Baker	JX517778	JX572564
*Erythrina lysistemon* Hutch	JF270764	JF265415
*Erythrina poeppigiana* (Walp.) Skeels	KJ012578	KJ082285
*Erythrina sacleuxii* Hua	KX146309	KU568087
*Erythrina sousae* Krukoff & Barneby	EU717411	EU717270
*Erythrina speciosa* Andrews	KX816365	AB045801
*Erythrina variegata* L.	KU587466	KU559206
*Erythrina velutina* Willd.	KY045858	JX856697
*Erythrina vespertilio* Benth.	JX850049	JX856700
*Erythrina zeyheri* Harv.	JX517714	JX572565
*Dysolobium grande* (Wall. ex Benth.) Prain	KX713094	KX527443

**Table 3:** Species of *Erythrina* cultivated in Naples according to the literature. Legend: ^1^ by the synonym *Erythrina laeta* Dehnh.; ^2^ as *Erythrina insignis* Tod.; ^3^ as *Erythrina Graefferi*’ (nomen nudum); ^4^ as ‘*E. crista-galli* L. var. *Andersonii*’ (nomen nudum); ^5^ as ‘*E. laurifolia*’ (i.e., *E. laurifolia* Jacq.); ^6^ as ‘*Erythrina hederaefolia*’ (i.e., *E. hederifolia* Spreng.); ^7^ as ‘*Erythrina umbrosa* H.B.’ (i.e., *E. umbrosa* Kunth); ^8^ as ‘*E. picta*’ (i.e., *E. picta* L.); ^9^ as ‘*Erythrina Pisonaja*’ (nomen nudum); ^10^ as *‘Erythrina bellengeri’* (nomen nudum). * Species nowadays cultivated at the Botanical Garden of Naples.

Species	Tenore (1807), [27]	Tenore (1813), [28]	Tenore (1819), [29]	Dehnhardt (1829), [41]	Dehnhardt (1832), [42]	Tenore (1839), [30]	Tenore (1840), [31]	Tenore (1842), [2]	Tenore (1845), [33]	Tenore (1848), [34]	Tenore (1855), [35]	Pasquale (1866), [36]	Pasquale (1867), [37]	Cesati (1867), [38]	Cesati (1869), [39]	Cesati (1872), [40]	Aliotta (1982), [43]	2017 *
*E. americana* Mill.					x ^1^												x ^1^	
*E. caffra* Thunb.													x, x ^2^					x
*E. corallodendron* L.	x	x		x	x				x				x					
*E. crista-galli* L.					x ^3^	x ^4^	x	x	x, x ^4^, x ^5^	x	x	x	x, x ^5^	x ^5^	x	x	x	x
*E. herbaceaea* L.		x		x	x				x^6^				x					
*E. humeana* Spreng.																	x	
*E. mitis* Jacq.									x ^7^									
*E. speciosa* Andrews			x	x					x				x					
*E. variegata* L.									x ^8^				x ^8^					
*E. velutina* Willd.									x				x					
Unidentified													x ^9^				x ^10^	

**Table 4:** Distribution of the sites in the city of Naples and their coordinates in UTM (Universal Transverse Mercator) extracted from Google Earth. See Figure 4 for the location on the map of Naples.

*E. crista-galli* Type Samples	Collection Sites	UTM Coordinates (m)	UTM Coordinates (m)
ECGPM	Piazza Municipio	436,948,76 E	4,521,091,25 N
ECGPV	Piazza Vittoria	436,041,31 E	4,520,442,85 N
ECGOB	Orto Botanico	437,741,73 E	4,523,616,01 N
***E. caffra* Type Samples**	**Collection Sites**	**UTM Coordinates (m)**	**UTM Coordinates (m)**
ECVC	Villa Comunale	435,218,25 E	4,520,535,67 N
ECVF	Villa Floridiana	435,073,49 E	4,521,358,05 N
ECPM	Piazza Mazzini	436,126,69 E	4,522,527,87 N
ECOB1	Orto Botanico	437,828,39 E	4,523,790,37 N
ECOB2	Orto Botanico	437,723,28 E	4,523,670,84 N

## Appendix A

**Selected examined dried specimens.** Legend: (NAP-Ten) = Herbarium *Neapolitanum*, Collection “Tenore”; (NAP-Guss), Herbarium *Neapolitanum*. Collection “Gussone Generale”, s.d. = sine die [without date], s.c. = sine collectore [without collector], s.l. = sine loco [without locality of gathering].

(A) ***E. caffra* Thunb.:** (1) Naples at the Villa Reale, April 1867, *s.c.* (NAP-Guss, sub *E. corallodendron*); (2) s.l., April 1884, *s.c.* (NAP-Guss, without species name); (3) Villa Bisignano, s.d., *s.c.* (NAP-Guss, sub *E. corallodendron*). (**B**) ***E. crista-galli* L.:** (1) s.l., s.d., *s.c.* (NAP-Ten, sub *E. laurifolia*); (2) Botanical Garden of Naples, s.d., *s.c.* (NAP-Ten, sub *E. poianthes*); (3) Villa Bisignano, s.d., *s.c.* (NAP-Guss, sub “*E. Andersonii* ?*”*); (4) s.l., s.d., *s.c.* (NAP-Guss, without species name); (5) Botanical Garden of Naples, s.d., *s.c.* (Nap-Guss). (**C**) ***E. herbacea* L.**: (1) s.l., s.d., *s.c.* (NAP-Ten, sub *E. hederaefolia* Spreng.); (2) Botanical Garden of Naples, 1878, *s.c.* (NAP-Guss, sub *E. hederaefolia* Tod.); (3) Garden of the Prince of Bisignano, s.d., *s.c.* (NAP-Guss).

## Appendix B

*Sequences for ITS2; matK and rbcL. ECOB: E. caffra* of Botanical Garden of Naples; *ECSA: E. caffra of* Manie van der Schijff Botanical Garden (Southafrica); ECPM: *E. caffra* of “Piazza Mazzini”, Naples; ECVF: *E. caffra* of “Villa Floridiana”, Naples; ECGPV: *E. crista-galli* of “Piazza Vittoria”, Naples; ECGPM: *E. crista-galli* of “Piazza Municipio”, Naples; ECGOB: *E. crista-galli* of Naples Botanical Garden.
